# Improving Pharmacists’ Awareness of Inadequate Antibiotic Use for URTIs through an Educational Intervention: A Pilot Study

**DOI:** 10.3390/healthcare10081385

**Published:** 2022-07-25

**Authors:** Sandra Magalhães, Marta Estrela, Tânia Magalhães Silva, Tainá Costa, Gabriella Crexinski, Catarina Simões, Margarida Pisco Almeida, Adolfo Figueiras, Fátima Roque, Maria Teresa Herdeiro

**Affiliations:** 1 iBiMED—Institute of Biomedicine, Department of Medical Sciences, University of Aveiro, 3810-193 Aveiro, Portugal; mestrela@ua.pt (M.E.); tania.m.silva@ua.pt (T.M.S.); 2 Department of Medical Sciences, University of Aveiro, 3810-193 Aveiro, Portugal; tainacosta@ua.pt (T.C.); gabriellacrexinski@icloud.com (G.C.); catarina.simoes@ua.pt (C.S.); 3 Department of Communication and Art/DigiMedia, University of Aveiro, 3810-193 Aveiro, Portugal; marga@ua.pt; 4 Department of Preventive Medicine and Public Health, University of Santiago de Compostela, 15705 Santiago de Compostela, Spain; adolfo.figueiras@usc.es; 5 Health Research Institute of Santiago de Compostela (IDIS), University of Santiago de Compostela, 15705 Santiago de Compostela, Spain; 6 Consortium for Biomedical Research in Epidemiology and Public Health (CIBER Epidemiology and Public Health—CIBERESP), 15706 Santiago de Compostela, Spain; 7 Research Unit for Inland Development, Guarda Polytechnic Institute (UDI-IPG), 6300-559 Guarda, Portugal; 8 Health Sciences Research Center, University of Beira Interior (CICS-UBI), 6201-001 Covilha, Portugal

**Keywords:** antibiotic dispensing, pharmacists, respiratory infections, online course, e-learning, website, mobile app, eHealthResp

## Abstract

The inadequate use of antibiotics led to the development of multi-resistant bacteria that are now causing millions of deaths worldwide. Since most antibiotics are prescribed/dispensed to treat respiratory tract infections, it is important to raise awareness among health professionals to optimize antibiotic use, especially within the primary care context. Thus, this pilot study aimed to evaluate pharmacists’ feedback about the eHealthResp platform, composed by an online course and a mobile application (app) to help in the management of upper respiratory tract infections (URTIs). Ten community pharmacists were invited to participate in this study, exploring the contents of the eHealthResp platforms and answering a content validation questionnaire composed by eight qualitative and thirty-five quantitative questions about the online course and mobile app. The eHealthResp platform is a comprehensive, consistent, and high-quality e-learning tool. Median scores of 5.00 were attributed to the course contents’ and clinical cases’ adequacy and correction. Most qualitative feedback was about completeness and objectivity of the course, and its usefulness for clinical practice. This study showed that eHealthResp has great potential as an e-health tool for the management of URTIs’ symptoms, which may ultimately aid in reducing inappropriate antibiotic use.

## 1. Introduction

The inadequate and overuse of antibiotics for decades led to an exponential increase in super-resistant bacteria that do not respond to current available antibiotics [1]. Presently, bacterial resistance to antibiotics is one of the major public health problems, with nearly 5 million deaths having been associated to this phenomenon in 2019 [2].

It is well known that bacterial resistance to antibiotics is a natural process [3]. However, the exponential growth of resistance rates that have been observed in the last decades is mainly caused by its misuse, particularly its overuse, in situations in which there is not a real evidence of the benefit of the use of antibiotics [4,5,6]. In fact, in outpatient care, there are more than 40% of inappropriate antibiotic prescriptions for upper respiratory tract infections (URTIs) [7], despite clinical guidelines advising other therapeutic approaches [8,9,10,11,12].

The majority of antibiotic prescriptions occurs within the primary care context, mainly for respiratory infections. In fact, respiratory tract infections are in the lead for doctor appointments in the context of primary health care [13,14]. The impact of antimicrobial resistance in public health worldwide and the fact that there are no new antibiotics ready to be used in clinics to which bacteria have not developed resistance yet is alarming. So, it is essential to develop innovative strategies to help health professionals optimizing the use of current therapeutic options, thus avoiding the prescription and dispensing of antibiotics in clinical situations where an antibiotic is not needed [14,15].

Educational interventions specifically designed for health professionals have proven to be efficient in improving practices for more informed prescription and the dispensing of antibiotics, thus leading to a decrease in the levels of these indicators [16,17,18]. These studies also show that active interventions appear to be more effective in reducing inappropriate antibiotic prescribing/dispensing practices, with digital health tools showing potential for improving both clinical care and patient outcomes [19]. The main goal of these interventions is to give health professionals, including pharmacists, the most recent scientific evidence and clinical practice guidelines on antibiotic usage. This will ensure they have all the required resources to optimize the use of antibiotics, avoiding the dispensing of these drugs in situations in which they are not needed and being aware of all consequences for public health of the overuse of antibiotics. Combining these educational interventions with e-health tools may be the best approach to promote good clinical practices and help patients with their clinical conditions, without putting the future of antibiotics at risk. In fact, it has been shown that clinical decision support systems can be a useful complementary tool to assist health professionals in their daily work, for instance for managing patient symptoms and dispensing proper medicines for conditions in which an antibiotic may not be necessarily regarded as first line treatment [16,20,21].

In light of all that has been stated above, we developed the eHealthResp project, which included an online course and a mobile app, to help pharmacists with the management of information and decision making regarding upper respiratory infections, with the aim of promoting a proper use of antibiotics [20].

The main goal of this study was to evaluate the feedback received from a group of pharmacists that used the eHealthResp platform, composed by the online course and the mobile app. This platform, composed of several presentations and algorithms, was created to support pharmacists on URTI management. This pilot study has allowed us to improve the eHealthResp platform, making its daily use more efficient and attractive for pharmacists.

## 2. Materials and Methods

### 2.1. Setting

The eHealthResp project was designed and developed by the research team with the aim to evaluate the effectiveness of e-health tools to help pharmacists in the management of symptoms associated with upper respiratory tract infections.

The eHealthResp project is a continual education program, composed by a digital-based educational intervention that includes (i) a 45 min session directed to pharmacists about antibiotic resistance; (ii) the eHealthResp online course, a user-friendly course directed at pharmacists, composed by 4 modules and 4 clinical cases about URTIs and (iii) the eHealthResp mobile app, a clinical decision support system.

The contents and the usability of the web platform where the online course is hosted was already validated by pharmacists in terms of contents by using a Delphi Method approach, as well as to its usability, through usability tests [20,21,22].

### 2.2. Pilot Study

A pilot study was conducted in a small group of community pharmacists from Portugal’s North Regional Health Administration (ARS-N), corresponding to the northern region of the country.

Ten pharmacists working in community pharmacies in the catchment area covered by the ARS-N were recruited via e-mail through a convenience sample to be part of this pilot study, by exploring the contents of both the online course and the mobile app. Signed consent was obtained from all participants, according to the general data protection regulation (GDPR), to allow for the use of their e-mail addresses to inform participants about the aims of the study and to access the online course, clinical cases, and a final evaluation, as well as to access the mobile app and create their own passwords. The anonymity of participants was safeguarded during all the process and participants were completely autonomous to explore the course and the app and give their evaluations.

### 2.3. eHealthResp Online Course

The eHealthResp online course (see Supplementary Material S1) is hosted in a Wordpress-based web platform and it was designed for pharmacists, aiming to assist in the management of upper respiratory tract infections. The course is composed of 4 modules with complete, state of the art information [21] about URTIs, namely an introductory module (module 0), common cold and flu (module 1), tracheobronchitis, pharyngotonsillitis, and rhinosinusitis (module 2), as well an acting protocol for pharmacists regarding URTIs (module 3). A final evaluation exam composed by 4 clinical cases with 2 multiple-choice questions each regarding the most probable diagnosis and the most adequate treatment is also part of the online course [20,21].

### 2.4. eHealthResp Mobile App

The eHealthResp mobile app (see Supplementary Material S2) is an e-health tool available for Android and iOS mobile systems, developed to aid community pharmacists to better manage patients’ symptoms associated with URTIs. It is a clinical decision support system, based on 4 algorithms associated with the most common symptoms of URTIs: cough, fever, sore throat, and nasal symptoms. Based on the presence/absence and intensity of the different symptoms, the app guides the most likely diagnosis and possible therapeutic or non-therapeutic approaches to treat it [22,23,24].

### 2.5. Content Validation Questionnaire

To validate eHealthResp (both the online course and the mobile app), every participant completed a questionnaire after finishing the online course and use the mobile app. The questionnaire (see Supplementary Material S3) has three main sections: (1) sociodemographic data (five questions on gender, age, education level, specialty, and years of experience); (2) four groups of closed questions for quantitative evaluation of the online course contents and clinical cases and the mobile app (classified in five parameters from “Totally disagree” to “Totally agree”), and (3) six open-answer questions to qualitatively evaluate the online course and the mobile app. The dimensions defined to evaluate the platforms were proposed among the research team members, who are experts in usability and user experience [22,24,25].

### 2.6. Statistical Analysis

Descriptive statistical analyses were conducted to demonstrate demographics and to quantitatively evaluate eHealthResp’s online course and mobile app contents. Results were expressed as mean (±standard deviation), median, and 1st and 3rd quartiles.

Qualitative analysis of data provided by pharmacists was also performed to better understand participants’ insights about the course and to clarify any problems or difficulties experienced.

The internal consistency of the questionnaire was estimated using the Cronbach’s α test, where a value ≥ 0.70 was defined as satisfactory reliability. To avoid α’s inflation, individual analyses were conducted for adequacy, correction, and completeness constructs [23].

### 2.7. Ethics Statement

This pilot study obtained ethics approval by the Guarda Polytechnic Institute’s Ethics Committee (code number: 7/2021). The compliance with the provisions of the GDPR-Directive 95/46/EC was ensured, guaranteeing the security, anonymity and confidentiality of all data provided by the participants. Participation in the study was voluntary and participants provided their informed consent before participation.

## 3. Results

All the ten community pharmacists enrolled in the pilot study answered a content validation questionnaire to evaluate both the online course and the mobile app.

### 3.1. Sociodemographic Characteristics of the Pharmacists

The first section of the questionnaire aimed to collect sociodemographic data from the participants (Table 1). The majority of the participants (70%, n = 7) were female and 30% were male (n = 3). The average age of the pharmacists was 48.1 (±9.41) years. Only two of the pharmacists (20%) had a post-graduation level education other than a master’s degree in pharmacy, and the average years of professional experience was 24.5 (±11.8).

### 3.2. eHealthResp Online Course Quantitative Evaluation

The questionnaire obtained a Cronbach α value of 0.966, with individual Cronbach α values of 0.909, 0.891, and 0.910 for adequacy, correction, and completeness dimensions, respectively. Table 2 shows the detailed evaluation of the online course, with median scores and correspondent 25 and 75 percentiles for each one of the analyzed parameters.

The median score of the general grade attributed to the online course by the study pharmacists was 5.00 out of 5.00, and all the modules and clinical cases received a median score of 5.00 out of 5.00 (Table 2).

### 3.3. eHealthResp Mobile App Quantitative Evaluation

The quantitative evaluation of the eHealthResp mobile app presented a median score of 5.00 out of 5.00, as depicted in Table 3. All the four analyzed parameters (format, utility, interest, and trust) presented a median score of 5.00 out of 5.00 (Table 3).

### 3.4. Qualitative Evaluation

Besides quantitative assessment, all the pharmacists enrolled in the online course and users of the mobile app answered some open questions, to give their own perception and opinion about the course and mobile app. These questions mainly focused on what they liked the most/least and what could be improved, as well as on the usefulness of the course and app.

Regarding the online course, most of the comments were about how the course is “complete”, “objective”, and “practical”, as well as “useful for our clinical practice”, helping to “refresh our knowledge so we can always give the best advice to patients”. Only two of the participants pointed out negative aspects of the course: “the slowness of the platform” and the “colors used in the slides”.

In what concerns the mobile app, the feedback was also positive, and most of the comments focused on the easiness of use and its practical nature. One of the users also referred to the app as “intuitive and fast in the clinical evaluation” and another pointed out that “it is helpful to ensure the diagnosis proposed was correct”, by using the app to confirm it.

Overall, pharmacists gave a remarkable evaluation to both the online course and mobile app and highlighted the importance of both e-tools, namely to optimize the use of antibiotics. One of the users reported that “a good training and proper diagnosis is essential so health professionals can help patients giving them the best medicines to specific symptoms, thus avoiding inappropriate antibiotic prescription”. Another user stated, regarding the mobile app, that “by using this tool to help in the diagnosis, one can evaluate the need for antibiotics, promoting a proper use of it and helping to slow down antibiotic resistance”.

## 4. Discussion

The eHealthResp online course and the mobile app are the most important part of an educational intervention designed for pharmacists, to promote good health practices among health professionals, and contribute to slow down the dissemination of multi-resistant strains of bacteria. Overall, the feedback received from the ten participants in this pilot study was highly positive, with median scores above four (out of five) for all evaluated points, showing the utility and interest of both the online course and the mobile app, and also reflecting its user-friendliness. The fact that the course can be easily accessed anywhere, from a smartphone, laptop, or tablet and that it does not have an expiration date, makes it even more attractive for pharmacists, since they can complete it at their own pace. Furthermore, both the online course contents and the mobile app can be accessed offline, which constitutes another major advantage in terms of its availability at any time and place.

The use of online learning tools for health professionals has been successfully used in different medical areas. In 2020, French et al. reported the results from a pilot usability study directed to general practitioners about the attention deficit hyperactivity disorder [25]. In that case, they had a sample of 10 physicians that evaluated the online intervention in terms of its usability, with 29 questions. The results showed that general practitioners considered the course helpful and were satisfied with the content and layout [25]. Another pilot study with 10 obstetrician–gynecologist resident physicians and 14 undergraduates assessed the usability of a reproductive health self-study website, comprising four online sessions [24]. The course was evaluated in terms of medical accuracy, ease of use, and overall usefulness, using the Likert scale, and the results unequivocally showed that the online platform is an acceptable way to learn. The biggest differences from these studies to this pilot study relies on the fact that we evaluated two e-tools (online course and mobile app) at the same time, instead of just one. In addition, regarding evaluation, we asked for an individual evaluation for each module, not only in terms of its usability but also completeness, correction, and accuracy. Furthermore, each question of the evaluation questionnaire of the eHealthResp platform (both the online course and the mobile app) had a 5-point Likert scale, which allowed for the feedback to be more precise than, for instance, the Yoost et al. study [24], where some questions only had a 3-point Likert scale, thus providing a less exact feedback.

One of the biggest strengths of this study is the way it was developed: the development process of both the course and the mobile app was totally iterative, which means it has been improved several times during the process of its development, resulting in the final pre-educational intervention product [22,23,24]. This constant improved is reflected in the good evaluation given by pharmacists.

The low number of participants is the main limitation of this study, since it restricts the amount of feedback received, which would be helpful for the continuous improvement of both the online course and the mobile app. However, it was extremely difficult to recruit pharmacists during this time, due to restraints associated with the COVID-19 pandemic, since during this time health professionals were overwhelmed with an excessive workload. Nevertheless, for this type of study, 10–12 participants were shown to be successfully used [26,27,28,29]. Though this questionnaire having not been content-validated may be regarded as a limitation, it was developed by a user experience and usability expert team, consequently reinforcing its high internal consistency.

The use of educational tools to improve antimicrobial stewardship has increased in the last years, with positive results regarding the decrease in prescription rates and inadequate prescriptions [17,30,31,32].

## 5. Conclusions

In conclusion, the results of this pilot study strengthened the easiness of use, comprehension, consistency, and quality of both the online course and the mobile app by pharmacists. Ultimately, the feedback provided highlights of both the platforms’ utility as a learning e-tool for management of URTIs symptoms. In the future, the eHealthResp platform will be a part of an educational intervention designed for a larger group of community pharmacists, focused on antimicrobial resistance, aiming to promote good practices for antibiotic dispensing and to ultimately reduce bacterial resistance rates.

## Figures and Tables

**Table 1:** Pharmacists’ demographic characteristics.

	N	%	AVERAGE	ST. DEV
**SEX**				
**MALE**	3	30.0%		
**FEMALE**	7	70.0%		
**AGE**			48.10	9.41
**EDUCATION LEVEL**				
**BACHELOR’S DEGREE**	7	70.0%		
**POST-GRADUATION**	2	20.0%		
**MASTER’S DEGREE**	3	30.0%		
**PHD**	0	0.0%		
**YEARS OF EXPERIENCE**			24.50	11.80

**Table 2:** Pharmacists’ quantitative evaluation of the overall parameters of the online course contents and clinical cases.

Modules	Parameters	Median (Q1, Q3)
Module 0 *Introduction*	Adequacy	5.00 (5.00, 5.00)
	Correction	5.00 (4.25, 5.00)
	Completeness	5.00 (5.00, 5.00)
Module 1 *Common cold and flu*	Adequacy	5.00 (4.25, 5.00)
	Correction	5.00 (4.25, 5.00)
	Completeness	5.00 (4.00, 5.00)
Module 2 *Tracheobronchitis, pharyngotonsillitis and rhinosinusitis*	Adequacy	5.00 (5.00, 5.00)
	Correction	5.00 (5.00, 5.00)
	Completeness	5.00 (5.00, 5.00)
Module 3 *Acting protocol*	Adequacy	5.00 (5.00, 5.00)
	Correction	5.00 (4.25, 5.00)
	Completeness	5.00 (5.00, 5.00)
**Clinical Cases**	**Parameters**	**Median (Q1, Q3)**
Clinical Case 1 *Flu*	Adequacy	5.00 (4.25, 5.00)
	Correction	5.00 (4.00, 5.00)
	Completeness	5.00 (5.00, 5.00)
Clinical Case 2 *Acute rhinosinusitis*	Adequacy	5.00 (4.25, 5.00)
	Correction	5.00 (5.00, 5.00)
	Completeness	5.00 (5.00, 5.00)
Clinical Case 3 *Acute* *pharyngotonsillitis*	Adequacy	5.00 (5.00, 5.00)
	Correction	5.00 (4.00, 5.00)
	Completeness	5.00 (4.25, 5.00)
Clinical Case 4 *Possible pneumonia*	Adequacy	5.00 (4.25, 5.00)
	Correction	5.00 (5.00, 5.00)
	Completeness	5.00 (4.25, 5.00)
**Online Course**	**Parameters**	**Median (Q1, Q3)**
	Format	4.50 (4.00, 5.00)
	Utility	5.00 (5.00, 5.00)
	Interest	5.00 (4.25, 5.00)
	Trust	5.00 (4.25, 5.00)
**Online Course General Grade**		**Median (Q1, Q3)**
		5.00 (4.00, 5.00)

**Table 3:** Quantitative evaluation of the mobile app.

Mobile App	Parameters	Median (Q1, Q3)
	Format	5.00 (4.00, 5.00)
	Utility	5.00 (4.25, 5.00)
	Interest	5.00 (4.25, 5.00)
	Trust	5.00 (4.00, 5.00)
**Mobile App General Grade**		**Median (Q1, Q3)**
		5.00 (4.00, 5.00)

## Data Availability

Not applicable.

## Supplementary Materials

The following supporting information can be downloaded at: https://www.mdpi.com/article/10.3390/healthcare10081385/s1, Supplementary Data S1: Online Course Platform; Supplementary Data S2: Mobile Application Platform; Supplementary Data S3: Content Validation Questionnaire
