# Supplementary material for: Improving Pharmacists’ Awareness of Inadequate Antibiotic Use for URTIs through an Educational Intervention: A Pilot Study
Source: Healthcare (Basel). 2022 Jul 25;10(8):1385. doi: 10.3390/healthcare10081385 (PMC9394361; doi:10.3390/healthcare10081385)
Supplement: Supplementary file 1 [file healthcare-10-01385-s001.zip › S1 - OnlineCourse_SuppMat.pdf]

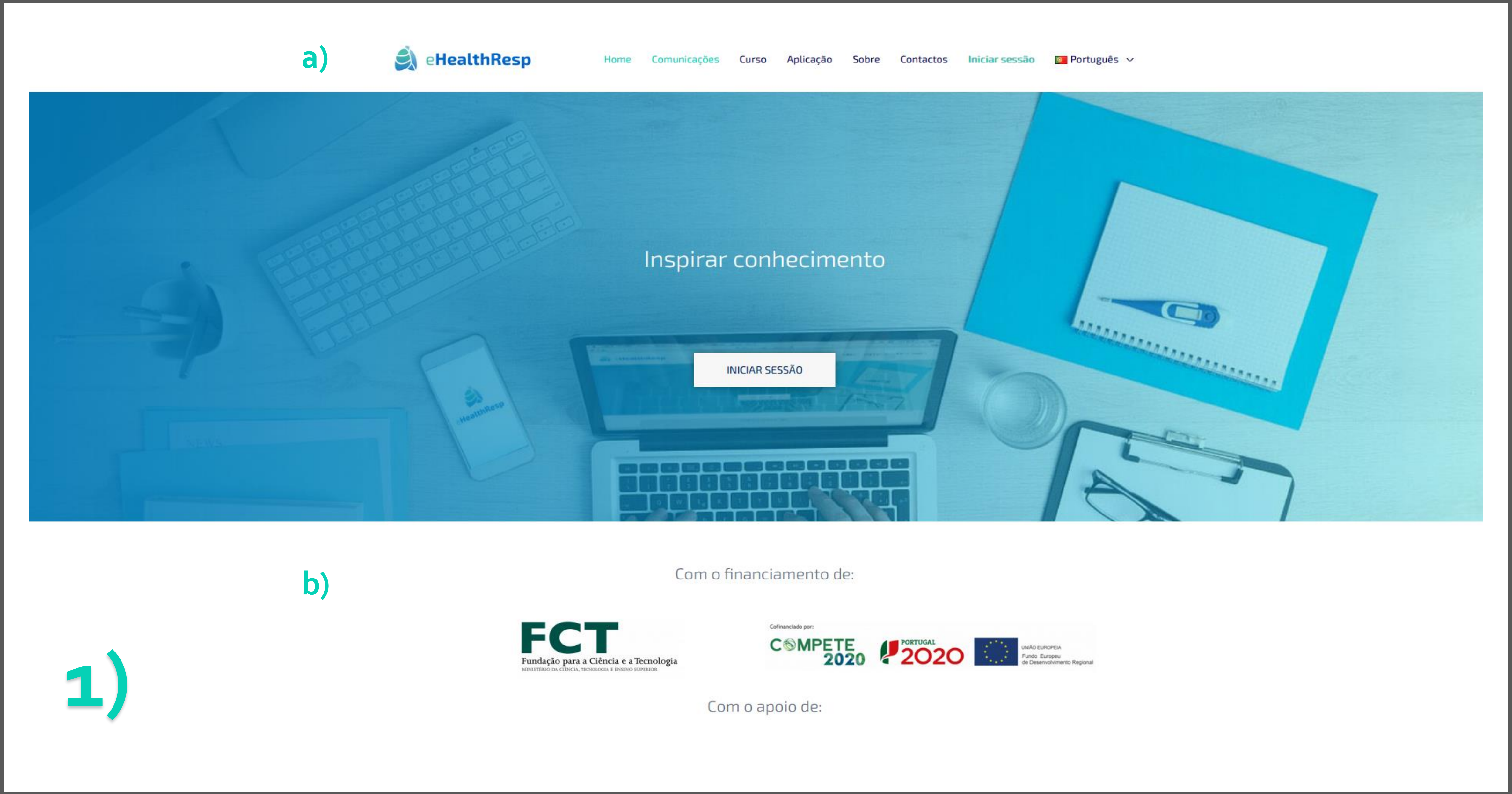

Homepage. a) website header; b) funding information

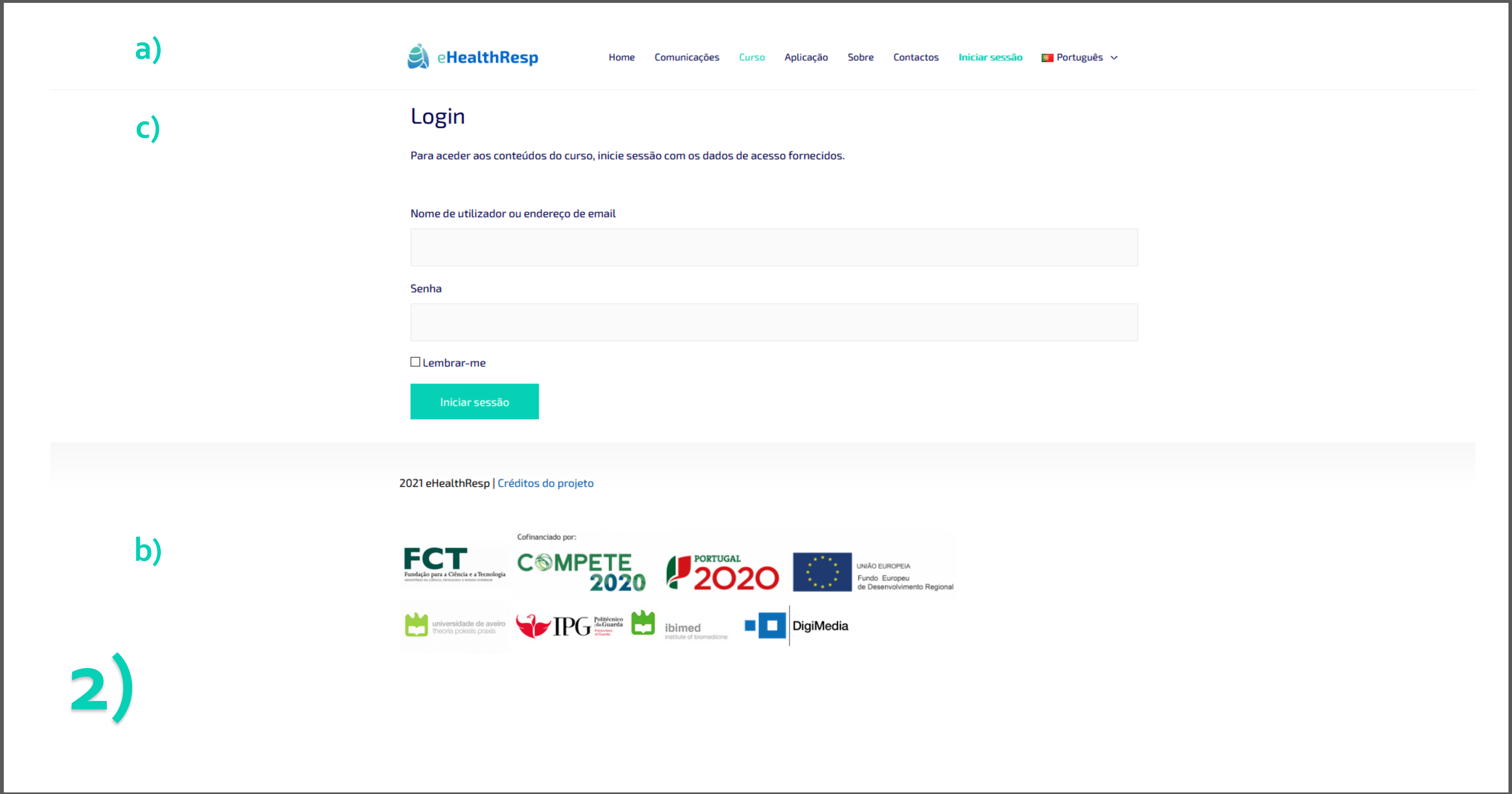

Login page. a) website header; b) funding information; c) login section

a)

eHealthResp

Home | Comunicações | Curso | Aplicação | Sobre | Contactos | Terminar sessão | Português

d)

Diagnóstico e terapêutica nas afeções respiratórias

Este curso é destinado a médicos. O objetivo é atualizar e consolidar conhecimentos no que concerne a afeções do trato respiratório superior. Através do (Link), terá acesso à aplicação móvel, que utiliza algoritmos de apoio à decisão clínica para uma avaliação adequada dos sintomas.

e)

15.38%

CONTINUAR

f)

Apresentação	
Introdução	1 de 1

Conteúdos	
Otite Média	1 de 6
Rinossinusite aguda	2 de 6
Faringoamigdalite Aguda	3 de 6
Bronquite Aguda	4 de 6
Pneumonia Adquirida na Comunidade	5 de 6
Infeções respiratórias em tempos de COVID-19	6 de 6

3)

Online course page. a) website header; d) module description; e) progress bar; f) online course modules

4)

a)

g)

h)

i)

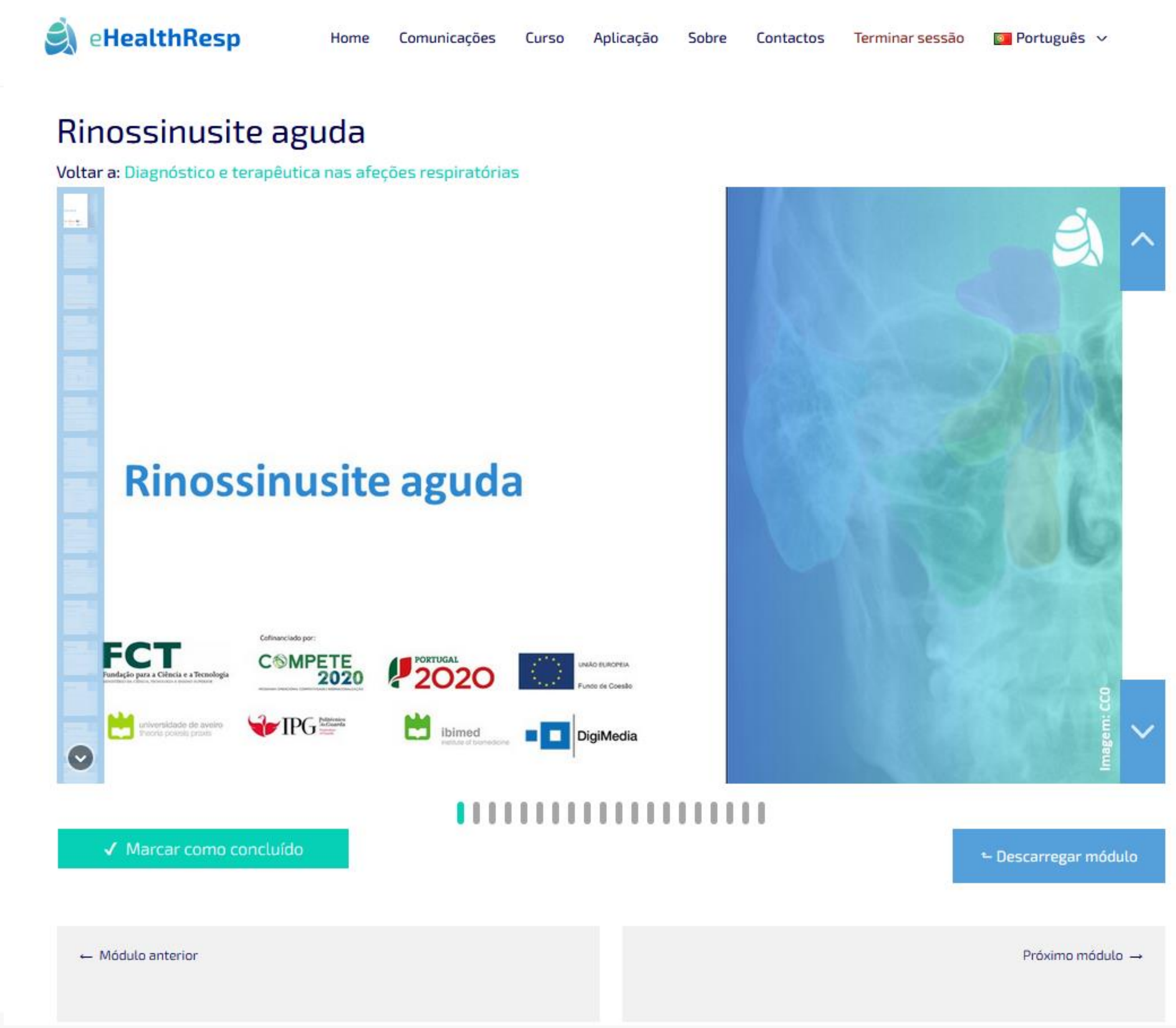

Module page example. a) website header; g) presentation; h) “mark as completed” and “download module” buttons; i) previous/next module buttons

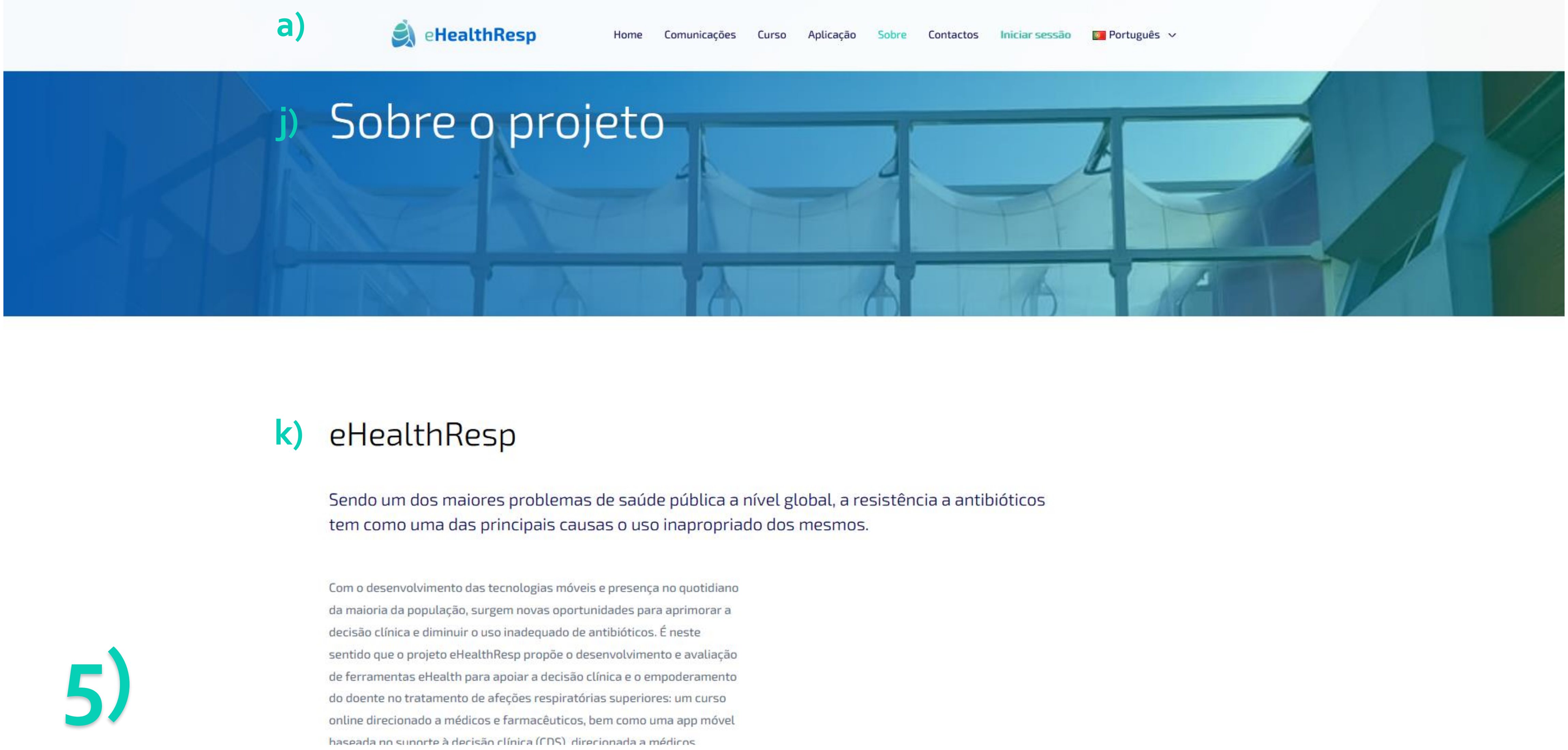

5)

Project information page. a) website header; j) “About the project”; k) eHealthResp project description

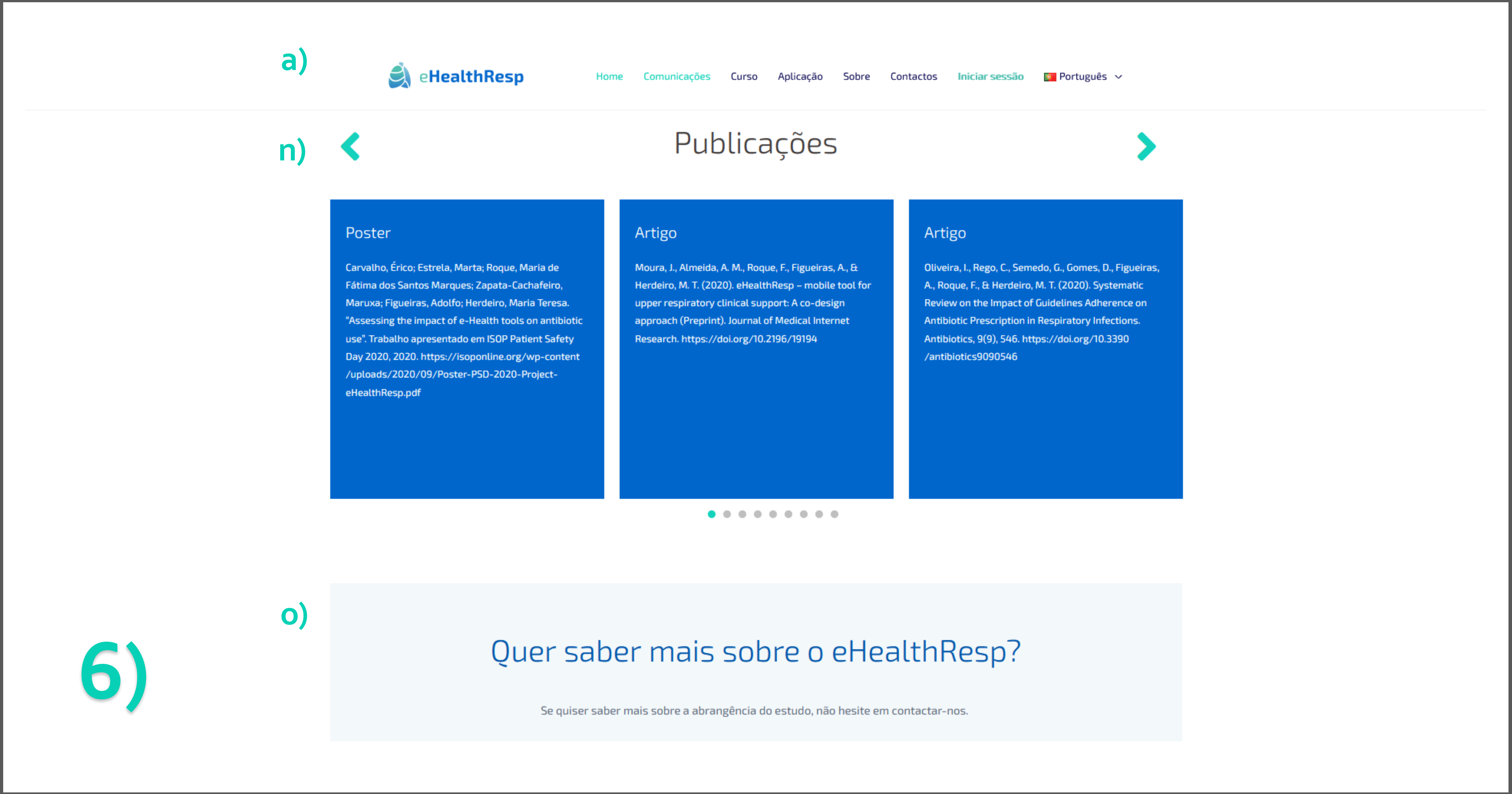

Publications page. a) website header; n) publications section; o) contact's section hyperlink

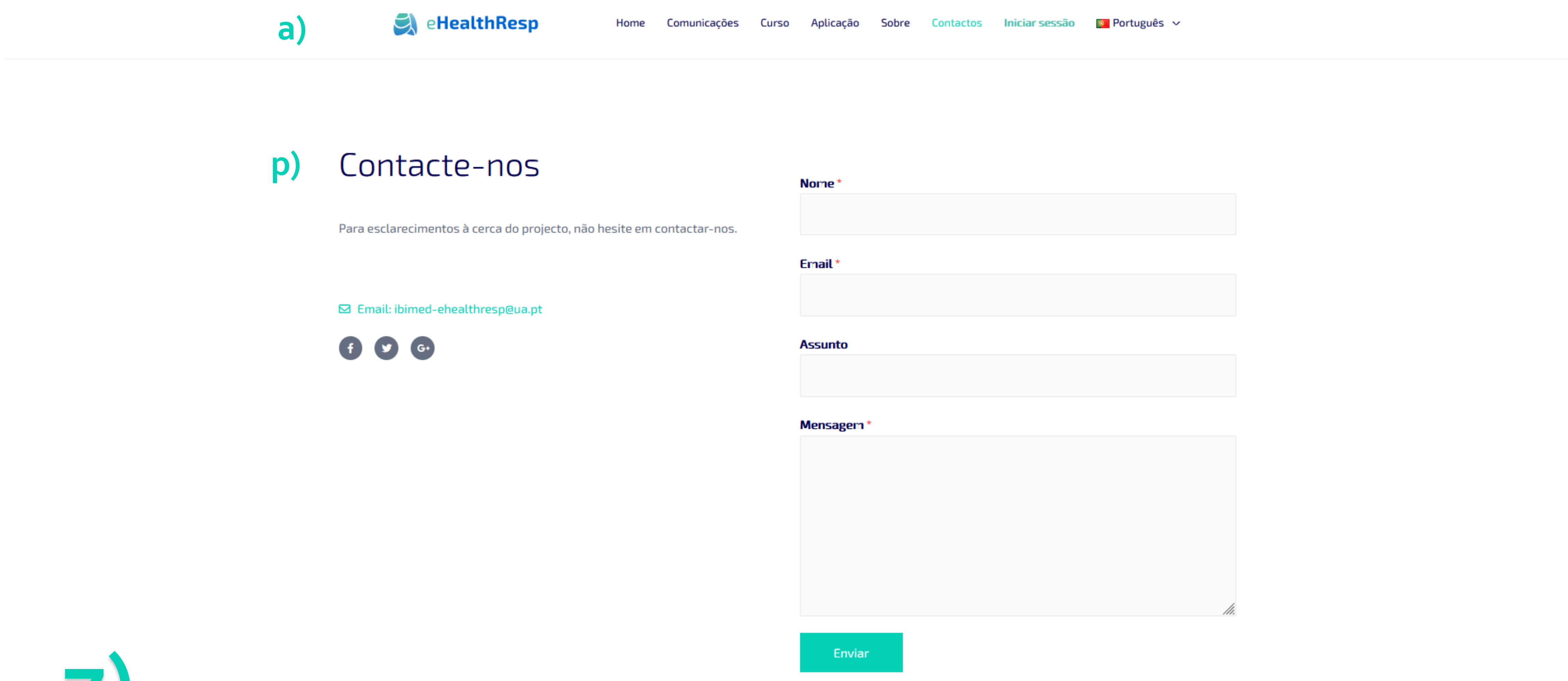

7)

Contacts page. a) website header; p) contact form

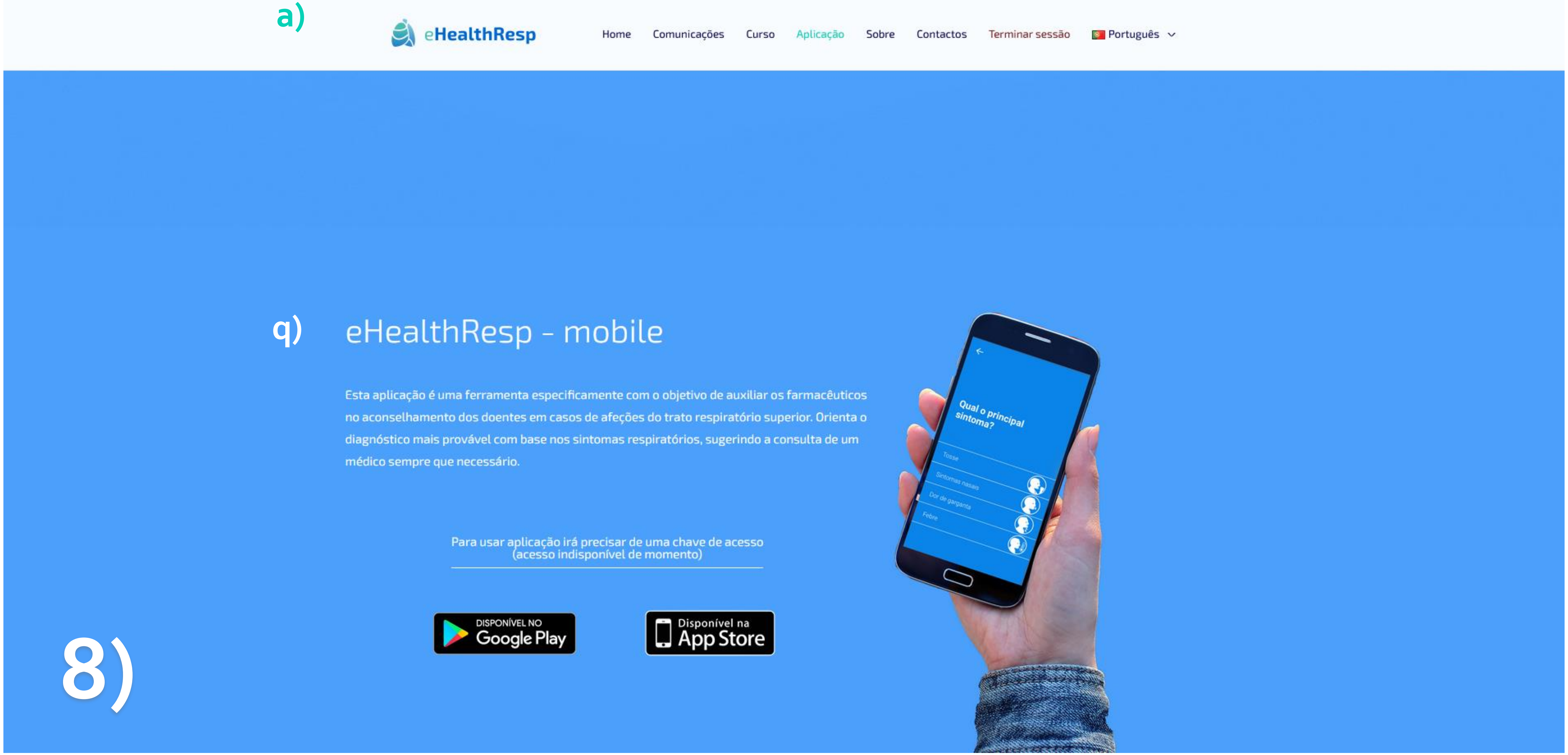

Mobile app page. a) website header; q) mobile app description and hyperlink to PlayStore (Android) and AppStore (iOS)
