# Supplementary material for: Improving Pharmacists’ Awareness of Inadequate Antibiotic Use for URTIs through an Educational Intervention: A Pilot Study
Source: Healthcare (Basel). 2022 Jul 25;10(8):1385. doi: 10.3390/healthcare10081385 (PMC9394361; doi:10.3390/healthcare10081385)
Supplement: Supplementary file 1 [file healthcare-10-01385-s001.zip › S2 - eHealthResp app_SuppMat.pdf]

About Us

Scientific authors

Adolfo Figueiras Guzmán[1]
Antonio Lirola Delgado[2]
Carlos Regueira Méndez[1]
Fátima Roque[3]
Maria Teresa Herdeiro[4]
Maruxa Zapata Cachafeiro[1]

Interface and usability

Ana Margarida Pisco Almeida[5]
Fátima Roque[3]
João Moura[4 5]
Maria Teresa Herdeiro[4]
Marta Estrela[4]

Graphic design

Claudia Ferreiro Vega[1]
João Moura[4 5]

Technical Authors

Verónica Rodríguez Cepeda[6]
José Pose Salgueiro[6]

More Information

1. Consortium for Biomedical Research in Epidemiology and Public Health (CIBER Epidemiology and Public Health-CIBERESP), University of Santiago de Compostela, Santiago

14:27

About Us

This work was financially supported by the project POCI–01–0145-FEDER-031678, funded by FEDER, through COMPETE2020 - Programa Operacional Competitividade e Internacionalização (POCI), and by national funds (OE), through FCT/MCTES.

v1.0.0

Authors Financing

What is the main symptom?

- Cough
- Nasal symptoms
- Sore throat
- Fever

14:27

What is your main suspicion?

- Acute otitis media
- Acute Rhinosinusitis
- Acute pharyngotonsillitis
- Tracheobronchitis
- Pneumonia

14:27 1.5 K/s 4G+ 63

Probable diagnosis

Acute otitis media

Treatment

Medication

- Treatment with anti-inflammatory and analgesic drugs to relieve pain
- Antipyretic if you have fever
- Use of nasal decongestants to facilitate drainage of the middle ear
- Vasoconstrictor nasal drops
- Ear drops (with prior assessment)
- Hydration
