# Supplementary material for: Improving Pharmacists’ Awareness of Inadequate Antibiotic Use for URTIs through an Educational Intervention: A Pilot Study
Source: Healthcare (Basel). 2022 Jul 25;10(8):1385. doi: 10.3390/healthcare10081385 (PMC9394361; doi:10.3390/healthcare10081385)
Supplement: Supplementary file 1 [file healthcare-10-01385-s001.zip › S3 - Questionnaire_SuppMat.pdf]

Questionário de avaliação do curso online e da app eHealthResp - Farmacêuticos

A resistência aos antibióticos é um dos principais problemas de saúde pública da atualidade e o uso inapropriado de antibióticos constitui um importante determinante no desenvolvimento de resistências bacterianas. Neste sentido, o projeto intitulado “Desenvolvimento e validação de ferramentas e-Health de apoio à decisão clínica e ao empoderamento do doente nas infeções respiratórias: ensaio controlado aleatório por clusters.” pretende avaliar a eficácia de ferramentas e-Health de apoio à decisão clínica e ao empoderamento dos doentes na gestão de situações clínicas do trato respiratório superior.

Foi desenhada uma intervenção educativa de reforço que inclui uma intervenção multifacetada em centros de saúde e farmácias, um curso online, um sistema de apoio à decisão clínica para os médicos e farmacêuticos, bem como uma ferramenta e-Health para os doentes. Este estudo apresenta, como principal objetivo, a validação global do curso *online* eHealthResp (constituído por uma série de apresentações e vários casos clínicos sobre infeções respiratórias superiores em adultos) e da aplicação móvel eHealthResp, por um grupo de médicos e farmacêuticos.

O questionário do presente estudo encontra-se dividido em três partes. A primeira parte, constituída por cinco breves questões, permitirá a recolha de dados sociodemográficos, como sexo, anos de experiência, nível de educação e a sua especialidade. A segunda parte contém quatro grupos de questões de resposta fechada, e a terceira parte consiste em quatro questões de resposta aberta, ambas com o objetivo de avaliar os elementos curso online e da *app* móvel.

Agradecemos desde já a sua colaboração. Caso seja necessário algum esclarecimento adicional sobre o estudo em questão, não hesite em contactar a equipa de investigação para o seguinte e-mail: ibimed-ehealthresp@ua.pt.

Muito obrigado.

A equipa eHealthResp

Este trabalho, com a referência PTDC/SAU-SER/31678/2017, foi financiado por fundos nacionais através da FCT/MCTES e pelo COMPETE-2020 -

Programa Operacional Competitividade e Internacionalização (POCI), na sua componente FEDER.

Este inquérito é anónimo.

O registo das respostas ao inquérito não contém qualquer informação sobre a sua identidade, excepto se alguma pergunta do inquérito solicitar alguma identificação e a fornecer.

Se usou um código para aceder a este inquérito este código não será guardado junto com as suas respostas. O código é gerido numa base de dados separada e apenas é utilizado pelo programa para registar que concluíu o inquérito. Não há forma de relacionar os códigos dos convidados a participar no inquérito com as respostas dadas.

Consentimento informado

O presente estudo enquadra-se no projeto "Desenvolvimento e validação de ferramentas *e-Health* de apoio à decisão clínica e ao empoderamento do doente nas infeções respiratórias: ensaio controlado aleatório por clusters.", com a referência PTDC/SAU-SER/31678/2017, financiado por fundos nacionais através da FCT/MCTES e pelo COMPETE-2020 - Programa Operacional Competitividade e Internacionalização (POCI), na sua componente FEDER, e apresenta como principal objetivo a validação de quatro algoritmos sobre infeções respiratórias superiores em adultos, associados ao curso *online* eHealthResp, para serem utilizados através de uma aplicação móvel (*App* eHealthResp).

O estudo dá cumprimento ao estipulado no Regulamento Geral de Proteção de Dados (RGPD) garantindo a segurança e confidencialidade de todos os dados facultados pelos participantes, assim como a garantia de que as respostas facultadas pelos mesmos nunca serão associadas com a sua identidade. Todos os dados recolhidos serão encriptados e arquivados eletronicamente, e só os investigadores do projeto terão acesso aos mesmos. As responsáveis pelo tratamento e recolha dos seus dados são Maria Teresa Herdeiro (iBiMED-UA) e Fátima Roque (IPG). Assegura-se ainda a total destruição de todos os dados após a apresentação do relatório sobre os resultados da implementação do projeto e finalização do mesmo, previsivelmente no final do mês de julho de 2022. Os resultados serão divulgados exclusivamente em contexto científico (em apresentações ou publicações), sendo tratados de forma agregada, e nunca individualmente. É possível desistir do estudo em qualquer momento.

Poderá exercer os seus direitos de acesso, retificação, eliminação, limitação e oposição, sempre que necessário, contactando por e-mail as investigadoras responsáveis pelo projeto, Maria Teresa Herdeiro (iBiMED-UA) e Fátima Roque (IPG), para o e-mail ibimed-ehealthresp@ua.pt (mailto:ibimed-ehealthresp@ua.pt). Poderá ainda contactar o Encarregado de Proteção de Dados da Universidade de Aveiro epd@ua.pt (mailto:epd@ua.pt). Caso seja necessário, poderá ainda apresentar reclamação à Comissão Nacional de Proteção de Dados em cnpd.pt (https://cnpd.pt).

✱Declaro que li e compreendi a informação que consta neste documento e que fui devidamente informado(a) e esclarecido(a) acerca dos objetivos e das condições de participação neste estudo e que, como tal:

- [x] ACEITO participar neste estudo
- [ ] NÃO ACEITO participar neste estudo

Dados demográficos

✱Sexo:

♀ Feminino	♂ Masculino

✱Idade:

❗ Verifique o formato da sua resposta.

✱Habilitações académicas:

❗ Selecione todas as opções que se apliquem

- [ ] Licenciatura
- [ ] Pós-Graduação
- [ ] Mestrado
- [ ] Doutoramento

✱Especialidade:

❗ Selecione todas as opções que se apliquem

- [ ] Doenças Infeciosas
- [ ] Medicina do Trabalho
- [ ] Medicina Geral e Familiar
- [ ] Medicina Interna

- [ ] Otorrinolaringologia
- [ ] Patologia Clínica
- [ ] Pneumologia
- [ ] Saúde Pública
- [ ] Outro:

✱Há quantos anos exerce a sua profissão?

❗ Verifique o formato da sua resposta.

Medidas de avaliação gerais do curso online eHealthResp

Por favor atribua uma classificação a cada um dos parâmetros abaixo apresentados, considerando a seguinte escala:

☆ Discordo totalmente ☆☆ Discordo ☆☆☆ Neutro ☆☆☆☆ Concordo ☆☆☆☆☆ Concordo totalmente

✱**Formato**: A apresentação destes conteúdos em formato digital é adequada.

★ ★ ★ ★ ★

✱**Utilidade**: Considero que a informação/conhecimento fornecida(o) pelo presente curso *online* é útil e que pode melhorar o processo de tomada de decisão clínica.

★ ★ ★ ★ ★

✱**Interesse**: Recomendaria o curso *online* eHealthResp a um colega.

★ ★ ★ ★ ★

✱**Confiança**: Tenho confiança nos conteúdos apresentados no curso *online.*

★ ★ ★ ★ ★

Medidas de avaliação dos módulos constituintes do curso online eHealthResp

Por favor atribua uma classificação a cada um dos parâmetros abaixo apresentados, considerando a seguinte escala:

☆ Discordo totalmente ☆☆ Discordo ☆☆☆ Neutro ☆☆☆☆ Concordo ☆☆☆☆☆ Concordo totalmente

*

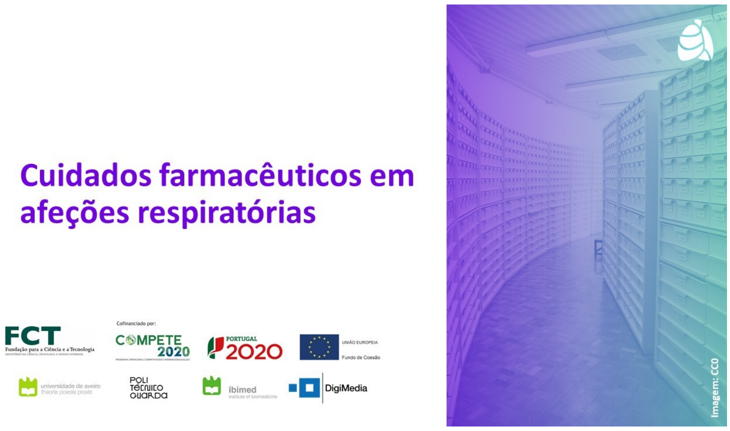

Módulo 0

Adequação: Considero que os conteúdos deste módulo são adequados.

★ ★ ★ ★ ★

***Correção**: Considero que os conteúdos deste módulo estão corretos e de acordo com a literatura.

★ ★ ★ ★ ★

***Completude**: Considero que os conteúdos deste módulo estão completos.

★ ★ ★ ★ ★

*

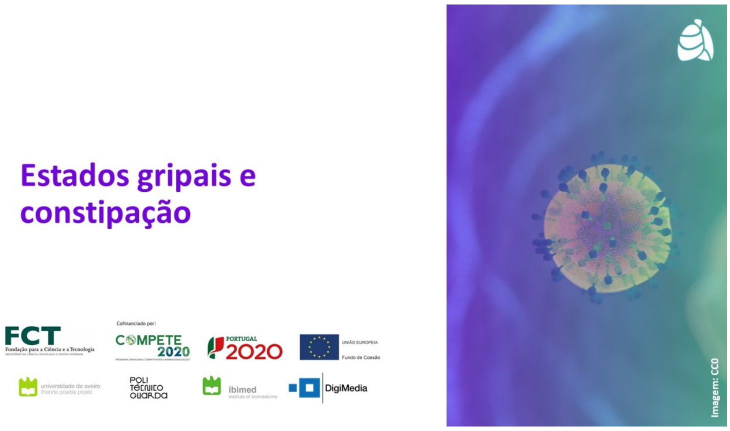

Módulo 1

Adequação: Considero que os conteúdos deste módulo são adequados.

★ ★ ★ ★ ★

✱**Correção**: Considero que os conteúdos deste módulo estão corretos e de acordo com a literatura.

★ ★ ★ ★ ★

✱**Completude**: Considero que os conteúdos deste módulo estão completos.

★ ★ ★ ★ ★

✱

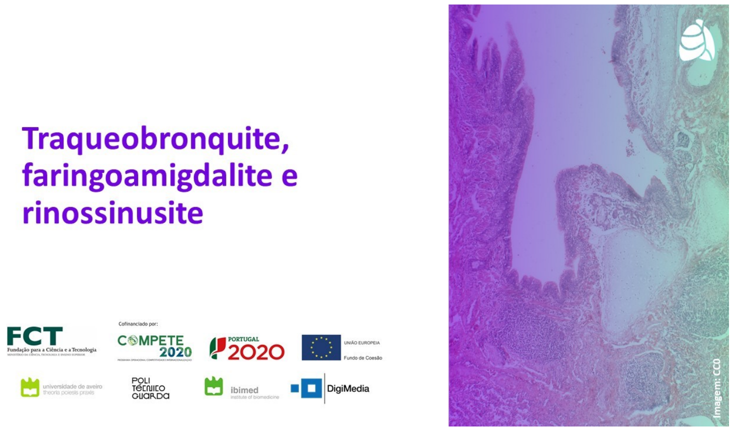

Módulo 2

Adequação: Considero que os conteúdos deste módulo são adequados.

★ ★ ★ ★ ★

✱**Correção**: Considero que os conteúdos deste módulo estão corretos e de acordo com a literatura.

★ ★ ★ ★ ★

✱**Completude**: Considero que os conteúdos deste módulo estão completos.

★ ★ ★ ★ ★

✱

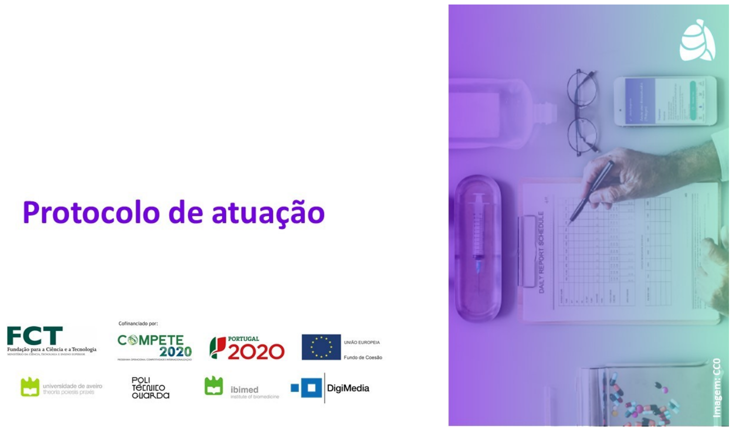

Módulo 3

Adequação: Considero que os conteúdos deste módulo são adequados.

★ ★ ★ ★ ★

✱**Correção**: Considero que os conteúdos deste módulo estão corretos e de acordo com a literatura.

★ ★ ★ ★ ★

✱**Completude**: Considero que os conteúdos deste módulo estão completos.

★ ★ ★ ★ ★

Medidas de avaliação dos casos clínicos constituintes do curso online eHealthResp

Por favor atribua uma classificação a cada um dos parâmetros abaixo apresentados, considerando a seguinte escala:

☆ Discordo totalmente ☆☆ Discordo ☆☆☆ Neutro ☆☆☆☆ Concordo ☆☆☆☆☆ Concordo totalmente

✱ Caso clínico 1

Adulto de 50 anos com febre desde ontem, calafrios, dores de cabeça e mialgias que aumentam. Sem sintomas nasais, sem tosse ou dor de garganta.

Adequação: Considero que os conteúdos, estrutura e apresentação deste caso clínico são adequados.

★ ★ ★ ★ ★

✱**Correção**: Considero que os conteúdos, estrutura e apresentação deste caso clínico estão corretos e de acordo com a literatura.

★ ★ ★ ★ ★

✱**Completude**: Considero que os conteúdos, estrutura e apresentação deste caso clínico estão completos.

★ ★ ★ ★ ★

✱ Caso clínico 2

Adulto de 38 anos, com muita congestão e obstrução nasal, com espirros esporádicos há mais de uma semana, não febril, com secreção nasal aquosa, perda de olfato e sem histórico de rinite alérgica.

Adequação: Considero que os conteúdos, estrutura e apresentação deste caso clínico são adequados.

★ ★ ★ ★ ★

✱**Correção**: Considero que os conteúdos, estrutura e apresentação deste caso clínico estão corretos e de acordo com a literatura.

★ ★ ★ ★ ★

✱**Completude**: Considero que os conteúdos, estrutura e apresentação deste caso clínico estão completos.

★ ★ ★ ★ ★

✱

Caso clínico 3

Jovem adulta de 22 anos de idade com febre de 38,5ºC, calafrios, dor de garganta intensa há 2 dias, principalmente ao engolir.

Adequação: Considero que os conteúdos, estrutura e apresentação deste caso clínico são adequados.

★ ★ ★ ★ ★

✱**Correção**: Considero que os conteúdos, estrutura e apresentação deste caso clínico estão corretos e de acordo com a literatura.

★ ★ ★ ★ ★

✱**Completude**: Considero que os conteúdos, estrutura e apresentação deste caso clínico estão completos.

★ ★ ★ ★ ★

✱

Caso clínico 4

Adulto 60 anos, fumador, com bronquite ocasional, apresenta muco e tosse produtiva intensa há uma semana. Febre de 39,2ºC com taquicardia e calafrios. Sem sintomas nasais ou dor de garganta.

Adequação: Considero que os conteúdos, estrutura e apresentação deste caso clínico são adequados.

★ ★ ★ ★ ★

✱**Correção**: Considero que os conteúdos, estrutura e apresentação deste caso clínico estão corretos e de acordo com a literatura.

★ ★ ★ ★ ★

✱**Completude**: Considero que os conteúdos, estrutura e apresentação deste caso clínico estão completos.

★ ★ ★ ★ ★

Medidas de avaliação gerais da aplicação móvel eHealthResp

Por favor atribua uma classificação a cada um dos parâmetros abaixo apresentados, considerando a seguinte escala:

☆ Discordo totalmente ☆☆ Discordo ☆☆☆ Neutro ☆☆☆☆ Concordo ☆☆☆☆☆ Concordo totalmente

✱**Adequação**: Considero que os conteúdos e informação fornecidos pela aplicação móvel são adequados.

★ ★ ★ ★ ★

✱**Correção**: Considero que os conteúdos e informação fornecidos pela aplicação móvel estão corretos e de acordo com a literatura.

★ ★ ★ ★ ★

✱**Completude**: Considero que os conteúdos e informação fornecidos pela aplicação móvel estão completos.

★ ★ ★ ★ ★

✱**Formato**: A apresentação destes conteúdos em formato digital é adequada.

★ ★ ★ ★ ★

✱**Utilidade**: Considero que a informação/conhecimento fornecidos/adquiridos pela presente aplicação móvel são úteis e que podem melhorar o processo de tomada de decisão clínica.

★ ★ ★ ★ ★

✱**Interesse**: Recomendaria a aplicação móvel eHealthResp a um colega.

★ ★ ★ ★ ★

✱**Confiança**: Tenho confiança nos conteúdos apresentados na aplicação móvel.

★ ★ ★ ★ ★

Questões de resposta aberta

✱Curso *online* eHealthResp:

O que mais gostou no curso?

O que menos gostou no curso?

Sente que beneficiou com o curso?

Em caso afirmativo, de que forma?

Em caso negativo, quais os motivos?

✱Aplicação móvel eHealthResp:

O que mais gostou na aplicação móvel?

O que menos gostou na aplicação móvel?

Sente que o uso da aplicação o(a) beneficiou a estabelecer um melhor diagnóstico?

Em caso afirmativo, de que forma?

Em caso negativo, quais os motivos?

Observações/comentários relativamente ao curso e/ou aplicação móvel eHealthResp:

* A resistência aos antibióticos é um dos principais problemas de saúde pública da atualidade e o uso inapropriado de antibióticos constitui um importante determinante no desenvolvimento de resistências bacterianas. Para combater este problema foi desenvolvida uma intervenção educativa que consiste no presente curso online e aplicação móvel eHeathResp, tendo como principal objetivo promover a redução da prescrição inadequada de antibióticos entre os profissionais de saúde.

eHealthResp

Considerando o objetivo do estudo acima mencionado, no geral pensa que esta intervenção educativa multifacetada poderá ser eficaz na melhoria da qualidade de prescrição de antibióticos?

Submeter
